# The ciliary protein Meckelin/TMEM67 interacts with HDAC6: possible implications for primary cilia stability

**DOI:** 10.1186/2046-2530-1-S1-P20

**Published:** 2012-11-16

**Authors:** R De Mori, B Illi, S Romani, S Valente, CA Johnson, A Mai, EM Valente

**Affiliations:** 1 Istituto Casa Sollievo della Sofferenza-Mendel, Italy; 2 Consiglio Nazionale delle Ricerche (CNR), Italy; 3 Universita La Sapienza di Roma, Italy; 4 Leeds Institute of Molecular Medicine, UK; 5 Universita' di Messina-Dipartimento di Scienze Pediatriche, Italy

## 

Ciliopathies are a group of autosomal recessive disorders, characterized by defect in central nervous system, that include several, partially overlapping, syndromes such Meckel-Gruber syndrome and COACH syndrome. Ciliopathies are caused by mutations in genes encoding protein of the primary cilium, as Jouberin and Meckelin (TMEM67). Meckelin is a receptor localized to the ciliary membrane and at the cell surface of polarized cells; it interacts with citoskeletal protein participating in ciliogenesis via remodelling of cytoskeleton. Histone Deacetylase (HDAC) 6 is a cytoplasmic deacetylase, that localize to microtubules in a variety of cultured cells, and participate in the deacetylation of the major component of the cilia axoneme, alpha-tubulin, a process that leads to reduced microtubule stability. Moreover, HDAC6 has also been implicated both in cilia resorption and disassembly, through its interaction with Aurora A kinase, that localize to basal bodies. In the present work we show that MKS3 interacts with HDAC6 in MKS3 overexpressing HEK293 cells and in ciliated IMCD3 cells. Moreover, we confirmed this interaction in mouse embryonic stem cells, used as an in vitro model of neurogenesis. In this cellular context, deacetylase activity assays performed on MKS3 immunoprecipitated complexes showed high HDAC activity, which was lost in the presence of a specific HDAC6 inhibitor. Altogether these results reveal an unpredictable interaction between Meckelin and HDAC6, shedding light on a putative, novel role of Meckelin in controlling ciliary microtubules acetylation/deacetylation and primary cilia stability.

